# Study of the synergistic activity between industrial yeast strains resistant to high temperature and ethanol concentrations and high fermentative capacity to produce ethanol

**DOI:** 10.1186/1753-6561-8-S4-P219

**Published:** 2014-10-01

**Authors:** Strohmayer Lourencetti Natália Manuela, Danieli Flávia, Valentini Roberto Sandro, de Lucca Gattás Edwil Aparecida, Soares Mendes Giannini Maria José, Zanelli Cleslei Fernando, Fusco Almeida Ana Marisa

**Affiliations:** 1 Department of Bioscience and Biotechnology applied to Pharmacy, Mycology Laboratory, UNESP, Araraquara, SP, Brazil; 2 Department Food and Nutrition, Fermentation Technology Laboratory, UNESP, Araraquara, SP, Brazil; 3 Department of Bioscience and Biotechnology applied to Pharmacy, Molecular Biology Laboratory, UNESP, Araraquara, SP, Brazil

## 

Fuel-ethanol fermentation process includes a reutilization of the yeast biomass, in which yeast cells are exposed to stress conditions as high temperature and increasing alcohol concentration. This environment may cause a significant delay in fermentation and drop in cell viability. The Bioen program (UNESP) conducted a study to select highly producing yeast strains in a Brazilian distillery. Two of these were selected when comparable to other well established commercial yeast strains: ZFC4 (ethanol best producer) and ZFD4 (most ethanol and temperature tolerant). Based on these data the aim of this work is to study genetic features associated with cell resistance to sustained stress of selected yeast strains in synergy with standards (PE-2, CAT-1 and SA-I) used in Brazilian distillery. The yeast strain was confirmed as *Saccharomyces cerevisiae *using PCR. The strains were screened together for growth of 25 at 44°C on plates containing 10% and 12% (v/v) ethanol and for fermentation assays. All of these demonstrated optimal responses to the high temperature and alcohol concentration and fermentative capacity when comparable to the same yeast strain singly. To gain insight of the cellular mechanisms of resistance to these stresses, global gene expression analysis of the selected strains will be performed.

